# Detection of Suicidal Ideation on Social Media: Multimodal, Relational, and Behavioral Analysis

**DOI:** 10.2196/17758

**Published:** 2020-07-07

**Authors:** Diana Ramírez-Cifuentes, Ana Freire, Ricardo Baeza-Yates, Joaquim Puntí, Pilar Medina-Bravo, Diego Alejandro Velazquez, Josep Maria Gonfaus, Jordi Gonzàlez

**Affiliations:** 1 Department of Information and Communication Technologies Universitat Pompeu Fabra Barcelona Spain; 2 Hospital de Día de Adolescentes Servicio de Salud Mental Consorci Corporació Sanitària Parc Taulí Sabadell Spain; 3 Departamento de Psicología Clínica y de la Salud Universitat Autònoma de Barcelona Barcelona Spain; 4 Department of Communication Universitat Pompeu Fabra Barcelona Spain; 5 Computer Vision Center Universitat Autònoma de Barcelona Bellaterra (Barcelona) Spain; 6 Visual Tagging Services Bellaterra (Barcelona) Spain

**Keywords:** social media, mental health, suicidal ideation, risk assessment, machine learning

## Abstract

**Background:**

Suicide risk assessment usually involves an interaction between doctors and patients. However, a significant number of people with mental disorders receive no treatment for their condition due to the limited access to mental health care facilities; the reduced availability of clinicians; the lack of awareness; and stigma, neglect, and discrimination surrounding mental disorders. In contrast, internet access and social media usage have increased significantly, providing experts and patients with a means of communication that may contribute to the development of methods to detect mental health issues among social media users.

**Objective:**

This paper aimed to describe an approach for the suicide risk assessment of Spanish-speaking users on social media. We aimed to explore behavioral, relational, and multimodal data extracted from multiple social platforms and develop machine learning models to detect users at risk.

**Methods:**

We characterized users based on their writings, posting patterns, relations with other users, and images posted. We also evaluated statistical and deep learning approaches to handle multimodal data for the detection of users with signs of suicidal ideation (suicidal ideation risk group). Our methods were evaluated over a dataset of 252 users annotated by clinicians. To evaluate the performance of our models, we distinguished 2 control groups: users who make use of suicide-related vocabulary (focused control group) and generic random users (generic control group).

**Results:**

We identified significant statistical differences between the textual and behavioral attributes of each of the control groups compared with the suicidal ideation risk group. At a 95% CI, when comparing the suicidal ideation risk group and the focused control group, the number of friends (*P*=.04) and median tweet length (*P*=.04) were significantly different. The median number of friends for a focused control user (median 578.5) was higher than that for a user at risk (median 372.0). Similarly, the median tweet length was higher for focused control users, with 16 words against 13 words of suicidal ideation risk users. Our findings also show that the combination of textual, visual, relational, and behavioral data outperforms the accuracy of using each modality separately. We defined text-based baseline models based on bag of words and word embeddings, which were outperformed by our models, obtaining an increase in accuracy of up to 8% when distinguishing users at risk from both types of control users.

**Conclusions:**

The types of attributes analyzed are significant for detecting users at risk, and their combination outperforms the results provided by generic, exclusively text-based baseline models. After evaluating the contribution of image-based predictive models, we believe that our results can be improved by enhancing the models based on textual and relational features. These methods can be extended and applied to different use cases related to other mental disorders.

## Introduction

### Background

Mental disorders are a serious health issue worldwide. According to the mortality data presented by the World Health Organization, the number of deaths because of suicide is equivalent to a person dying every 40 seconds [1]. Considering that the signs and symptoms of these disorders have been proven to be traceable on social media, scientists have started to work on the development of automated methods to detect signs and symptoms of these conditions [2-5] by addressing the importance of early detection [6,7].

State-of-the-art approaches for the detection of mental disorders in social media involve the use of machine learning techniques mainly based on textual features extracted from the posts [8-11]. These models require the previous acquisition of annotated data, which are extracted from a selected social platform. Considering this as a classification task, which manipulates sensitive data [12], a proper annotation process is required to obtain a reliable dataset. This has become one of the main challenges because diagnosing mental disorders involves the intervention of specialized and experienced clinicians who follow strict screening proceedings [11].

The methods used for analyzing user-generated data related to suicide focus on tracking social networks at a post level, *that is*, a tweet [13,14], or at a user level, *that is*, a sample of their tweets or posts [15]. The latter is more related to risk assessment, as more data from a single user can be explored. In terms of the type of information extracted and explored, state-of-the-art approaches perform an exhaustive analysis of textual information contained in posts. This has been proven to be relevant for screening and risk assessment tasks [16]. The methods applied often consider bag of words (BoW) models, topic models, lexicons, sentiment analysis tools, and readability and syntactical analysis features [5,15,17]. The most recent work with deep learning approaches consists of exploring sequence models encoding vector representations of terms known as word embeddings [3,11,15].

There are a few approaches that analyze features containing behavioral and relational information. Colombo et al [18] examined the connectivity and communication of users with suicidal ideation on Twitter based on the evaluation of retweets, whereas the work of De Choudhury et al [16] on Twitter defines an egocentric social graph for depression detection. However, these studies do not evaluate the performance of relational elements combined with image-based data.

Regarding the use of visual information from posts shared in social networks to address mental disorders, the closest approaches to our goal are methods for personality prediction [19], and a few recent approaches address self-harm, depression, and anxiety [20-22]. We believe that our study is the first image-based approach for suicide risk assessment on social media at the user level.

When we refer to suicide-related collections, to the extent of our knowledge, no previous publications have been reported on the generation of a suicide-related dataset built over Twitter where the annotation process involves expert clinicians labeling tweets according to the presence of suicidal thoughts focused on two types of control groups. Furthermore, our annotation was performed at the user level after having processed and analyzed sequences of posts within a period of a year. This allowed us to explore changes in time, as it has been proven to be useful for behavior analysis [23] and for the evaluation of early risk detection methods for depression [7,24].

### Objectives

This study addressed the detection of mental health issues on social media, with suicide risk assessment as our use case. Our main contributions are listed as follows: (1) we defined a methodology to generate a reliable Twitter dataset for suicide risk assessment, which is also the first user-level dataset of this type dedicated to posts in Spanish; (2) we presented a method to obtain a subset of the user tweets related to a specific topic, in this case, *suicidal ideation*; (3) we generated models that explore the impact of not just relational and behavioral factors but also elements identified by specialists during consultations, which have been mapped to social networks; (4) we developed image-based predictive models to detect suicidal ideation; (5) we integrated the previous elements into a method that combines multimodal data to build predictive models that address the detection of mental health issues using cross-platform information (Reddit, Instagram, and Twitter); and (6) we refined the evaluation process of predictive models for mental health issues by considering 2 different types of control groups within the social media context: users with posts that might not use terms related to mental conditions (generic control cases) and users who make use of terms related to mental disorders (focused control group).

## Methods

### Data Collection

In this section, we described the methodology designed to generate a reliable Twitter dataset for suicide risk assessment. We selected Twitter as our main data source, as it has been proven to be suitable for analyzing mental disorders on social media [3,13,18,25-27], including suicidal ideation. We also highlighted the following aspects that this platform offers for our research: (1) the possibility of selecting posts in multiple languages; (2) the availability of relational and behavioral factors; and (3) the provision of a set of chronologically ordered posts from each user, which allowed us to do a cumulative analysis of the data referring to suicidal ideation.

#### Combining Reddit and Twitter for Gathering Suicide-Related Data

Before starting the crawling process on Twitter, we elaborated a list of suicide-related sentences to build a first filtering approach to obtain tweets coming from users at risk. In doing so, we started by collecting a sample of 500 titles of posts published in Reddit’s Suicide Watch forum [28]. These posts were mostly written by users with suicidal ideations, so their titles can be considered to be suicide-related sentences.

These phrases were then translated to Spanish and reviewed by clinic psychologists. Some sentences were added, discarded, and/or adapted by the clinicians. We kept 454 phrases after the analysis by experts. Then, based on their relevance and link to suicide risk factors [29], a subset of 110 phrases were selected for use as search terms on Twitter. A total of 98,619 tweets containing these phrases were extracted for a year, that is, from December 21, 2017, to December 21, 2018. These tweets corresponded to 81,572 Twitter users, with 9559 users having more than one tweet matched with the search terms. At the same time, for all users, we extracted all their tweets posted within the same search period.

#### Two-Level Annotation Process

##### First Level

In total, 2 labeling stages were followed to identify users with suicidal ideation. As our intention was to follow a manual labeling process done by clinicians, we selected a random sample of 1200 users among those who had at least two tweets matching our search phrases. The user names were anonymized, and 3 tags were defined for labeling purposes: (1) *control—*defining users who on their tweets did not seem to manifest suicidal ideations, users who did not refer to their own conditions, and users who were reporting news or opinions regarding suicide; (2) *suicidal ideation risk—*labeling users who, judging by their writings, seemed to present suicidal ideation signs; and (3) *doubtful*—dedicated to cases where psychologists were not sure about labeling users within any of the other categories. At the first labeling stage, a clinician specializing in this topic was asked to classify users within these 3 categories based only on the tweets containing the suicide-related keywords. After the labeling process, 73.8% (885/1200) of users were classified as control cases, 9.6% (115/1200) were classified as suicidal ideation risk cases, and 16.7% (200/1200) fell within the doubtful category.

##### Second Level: Short Profile Version

A second labeling process was followed for the users tagged as suicidal ideation risk cases. We analyzed more of their collected tweets to confirm their labels. Annotators noticed that there was a high number of tweets that were not related to suicidal ideation and even sometimes no tweets related to suicide were caught in the sample. To address this issue, we developed a classifier at a tweet level to distinguish tweets containing signs of risk from those that were not related at all with suicide. Thus, we could provide the second annotator a summarized version of a user profile, which we call short profile version (SPV), that contained mainly tweets related to suicide and its risk factors.

We built a binary classifier distinguishing 2 classes: (1) *suicidal ideation–related tweet* and (2) *control tweet*. To train the model, we chose as instances for the *suicide tweet* class the tweets of users tagged as *suicidal ideation risk* cases (513 tweets) and 346 phrases evaluated by the clinicians. For the *control tweet* class, we selected an equally proportional set of random tweets related to other topics, using Twitter’s Sample Tweets application programing interface (API) [30]. A BoW model with 1 to 5 grams was generated, and after applying principal component analysis and logistic regression analysis, we achieved F1=0.90, precision (Pr)=0.91, and recall (R)=0.89, with a 10-fold cross-validation procedure. This is considered as our *short profile version classifier* (SPVC).

The SPVC was applied to every tweet of the profile of all users labeled as *suicidal ideation risk* and, for each user, we selected the top 15 *suicide-related tweets* with the highest predicted probability values given by the SPVC. We considered these tweets as the sample to be evaluated by 2 additional annotators: a specialized clinician and a nonspecialized annotator. This second annotator was given detailed instructions and information on risk factors related to suicide. The annotators at this stage were asked to classify users into 2 categories: (1) *suicidal ideation risk* or (2) *control*, now having more information about each user. Once the second annotation process was completed, we only retained the positive cases (n=84) on which both annotators agreed, corresponding to 89% (84/94) of all the cases labeled as suicidal ideation by at least one of the annotators in this second stage. Thus, we ensured that the users labeled as *suicidal ideation risk* were classified under the agreement of 3 annotators at different stages.

We defined 2 different control groups with the same size as the *suicidal ideation risk* class:

- *Focused control group*: users writing suicide-related keywords in a non–suicidal ideation risk context, that is, users who trivialize about suicide, news reports, and information regarding the topic; or users who simply manifest their support or opinions to people at risk. Identifying these users is challenging for classification systems but is key in reducing false-positives. These users were chosen at random among the users labeled as control cases during the first annotation process.
- *Generic control group*: a set of Twitter users who might not necessarily use terms related to suicide. These users were selected randomly using the Sample Tweets API [30] and anonymized as had been done for the other groups.

For both the control groups, the second annotation process was followed to discard possible cases of users at risk within these samples.

### Combining Multimodal Data for Detecting Suicide Risk

We proposed a method that given the profile of a user: (1) it uses a text-based model, described previously as the SPVC, which selects a subset of relevant tweets related to suicidal ideation. The set of tweets for which the SPVC provides a score over a given threshold is retained in SPV itself; (2) mostly from the outputted SPV, it extracts a set of relational, textual, behavioral, lexical, statistical, suicidal ideation–related, and image-based features from the content and metadata of the tweets; and (3) it builds and evaluates different predictive models resulting from the combination of these features. Our features are organized into 3 different groups: (1) *BoW or n-grams and word embeddings* as a representation of textual features; (2) a set of features known as *social networks and psychological (SNPSY) features* containing a group of relational, behavioral, lexical, sentiment analysis, and statistical features, in addition to a set of features that attempt to map to the social media context certain signs and symptoms, which are usually considered by clinicians at the time of screening; and (3) an *image-based score*.

#### Features Definition

##### Generic Text-Based Features

###### Bag of Words and N-Grams

These are features that have been used to address similar tasks, such as depression detection and eating disorders screening [7,31]. These models represent terms or sequences of terms (n-grams) based on their frequencies on the documents analyzed. In our case, each user was represented by a document consisting of the concatenation of the text of all their tweets. Afterward, we used the *Scikit-learn* [32] Python library: *TfIdfVectorizer* to generate a *tf.idf* representation of 1 to 5 grams at the word level. A set of Spanish stop words were considered to build this representation [33]. These features are referred to as BoW features in further sections. We also used *ekphrasis* [34] as a text preprocessing tool to replace generic tag elements such as money, phone numbers, digits, hashtags, and emoticons. We also removed the n-grams that appeared in less than 5% of the documents to reduce the feature space. This is done considering that the features’ number is given by the size of the vocabulary of all the writings of the users, and in Twitter, we found cases in which terms are misspelled or elongated (ie, *hellooooo* instead of *hello*); therefore, we avoided having multiple representations for the same term assuming that each spelling mistake and elongation is different and less likely to be repeated over most of the documents.

###### Word Embeddings

Word embeddings are representations of textual terms as vectors of real numbers. Words that are semantically related have a similar representation over the vector space. Most of the recent predictive models dealing with textual features using deep learning techniques make use of word embeddings to represent terms. The sequences of these representations are fed as inputs to train the predictive models. These types of representations have been recently used in state-of-the-art approaches to address suicide risk assessment [3,11]. We made use of word embeddings previously learned over a dataset with 2 million Spanish tweets [35].

##### Social Networks and Psychological Features

These consist of a group of features based on generic lexicons [36], statistics measured from the users’ writings, information of interest for clinicians regarding the behavior of users in time, the users’ social network (relational features) [16], and n-grams lexicons, which include terms referring to suicidal ideation or suicide risk factors (we referred to these features as suicide-related lexicon features). Each of these types of features is described in the next subsections.

###### Behavioral Features

These features are based on the information extracted from the metadata of tweets. Here, we measured the behavior of users based on their activity within certain periods, which are defined at different granularity levels. These features are detailed in Table 1.

The intention of the sleep time tweets ratio (STTR) and the daytime tweets ratio (DTTR) features is to identify the differences between control users and users at risk regarding the periods of the day on which they post. Considering that our data collection is delimited by language but not by location, that the posting time provided for a tweet is in coordinated universal time and not the time of the user location, and that not enough information from our data was found to automatically identify the location of all the users, we defined an approach to address this issue. As explained in Equation 1, a day was divided into 8 fixed time slots of 3 hours each. Afterward, we assumed that an average user had at least around 6 hours of sleep time, and within this 6-hour period, a smaller number of tweets would be created compared with the rest of the day, so we counted the number of tweets (t) created within each 3-hour time slot for all the tweets of the SPV of a user. Next, for each user, we calculated the sum of the number of tweets within each pair of continuous time slots and selected the minimum score obtained by all the pairs. We also assumed that the first and last slots can be continuous. Finally, this value was normalized according to the total number of tweets of the full profile of the user (T). This feature was considered as the STTR:

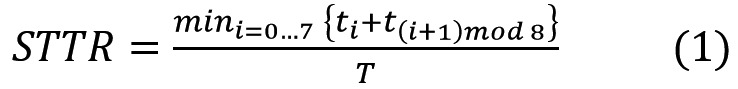



The DTTR was given by the difference between 1 and the sleep time ratio: that is, DTTR=1−STTR. It is important to recall that for the measurements that refer to a bigger granularity such as weekdays, weekends, and months, the impact of time difference is not as big as for features based on day periods.

###### Tweets Statistics

This group refers to 5 types of features that correspond to statistical measures calculated from the tweets of users. We considered elements such as the number of tweets created and their length and the number of tweets that were retained for each user at the SPV in relation to the total number of tweets posted. These features are described in Table 2.

###### Relational Features

These are informative features regarding the relationships and interactions between users. Elements such as the count of retweets and favorites received and given by the users can provide insight on the social support they receive, along with information regarding the number of followers and users followed, as previously considered for depression screening [16]. Table 3 describes the relational features extracted for our evaluation.

**Table 1:** Description of behavioral features.

Feature	Description	Source
Working week tweets count ratio	Total number of tweets on weekdays (Monday to Friday) normalized by the total amount of tweets	SPV^a^ tweets
Weekend tweets count ratio	Total number of tweets on weekend days (Saturday and Sunday) normalized by the total amount of tweets	SPV tweets
Median time between tweets	Median of the time (in seconds) that passes between the publication of each tweet	SPV tweets
Sleep time tweets ratio	Ratio of tweets posted during the inferred sleep period of the user	Full profile tweets
Daytime tweets ratio	Ratio of tweets posted during the period the user is usually awake	Full profile tweets
Normalized tweet count per quarter (4 features)	Number of tweets posted by the user within each quarter of the year, normalized by the total amount of tweets generated by the user during the year	SPV tweets

^a^SPV: short profile version.

**Table 2:** Description of features based on tweet statistics.

Feature	Description	Source
Suicide-related tweets ratio	Ratio of tweets retained by the SPVC^a^ over all the tweets of the full profile	SPV^b^ and full profile tweets
Median SPVC score	Median of the scores obtained by the tweets that are part of the SPV after applying the SPVC	SPV tweets
Median tweet length	Median length of all the user tweets (word level)	SPV tweets
Number of SPV tweets	Number of tweets	SPV tweets
Number of user tweets	Number of tweets posted by the user since the creation of the account	Tweet metadata

^a^SPVC: short profile version classifier.

^b^SPV: short profile version.

**Table 3:** Description of relational features.

Feature	Description	Source
Followers number	Number of followers	Tweet metadata
Friends number	Number of accounts followed by the user	Tweet metadata
Favorites given	Total number of favorites given by the user	Tweet metadata
Median favorites count	Median of the favorites received by the user	SPV^a^ tweets
Median retweets count	Median of the retweets received by the user	SPV tweets

^a^SPV: short profile version.

###### Lexicons and Suicide Risk Factors Vocabulary

The use of lexicons has been proven to be successful for tasks dedicated to screen mental disorders [9]. For our approach, we counted the frequency of words belonging to all the categories of the Linguistic Inquiry and Word Count (LIWC) 2007 Spanish dictionary [36,37] normalized by the size (in number of terms) of the concatenated writings of the users. It is important to recall that LIWC also contains categories that identify syntactical elements such as verbs, nouns, adverbs, and pronouns, among others. To this dictionary, a group of other categories was added containing vocabulary and up to 3-gram phrases that could be mapped to suicide-related terms and risk factors such as suicide methods; terms referring to self-injuries; explicit suicidal ideation references; self-loathing terms; words that might imply disdain, insomnia, and fear; and possible references to previous suicide attempts, experiencing racial or sexual discrimination, eating disorders, substance abuse, bullying, lack of social support, and family and money issues, along with vocabulary that might imply that some sort of discrimination or abuse has been suffered, that someone close has died from suicide, and even vocabulary regarding the lack of spiritual beliefs, as religion is considered to be a protective factor for screening tasks [29]. The terms and phrases selected for these categories were based on manually mapping common terms and phrases seen in a sample of tweets labeled as suicide related during the first labeling process with the assessment of clinicians. These features were calculated using SPV.

###### Sentiment Analysis

We obtained a score for each tweet in terms of its polarity. For this purpose, we used *senti-py* [38], trained on Spanish texts from different sources, including Twitter. It is based on a BoW model with an intermediate feature selection process. To obtain a score per user, we calculated the median of the scores of all the tweets from the SPV.

##### Image-Based Feature

We followed the methodology proposed in the study by Rodriguez et al [39], where a method for inferring the personality under the OCEAN model was presented. In this sense, we created a classifier trained on images extracted from Instagram using a subset of the phrases and keywords used in the data collection process for Twitter. These images were considered suicide related, whereas a set of unrelated images was considered as our control cases. Afterward, this first model was applied to each of the images extracted from the users’ tweets of our dataset. To create this model, we used 90,000 images for training and 60,000 images for validation. To obtain a single score per user (*images user score*), the average of the individual scores of the images of each user was considered as the user’s aggregated score.

To define the image classifier, we used a convolutional neural network (CNN) [40] because CNNs are especially suited for image data. There are several variants of this type of network, but the most popular ones are based on the residual networks introduced by He et al [41]. They used skip connections between layers that force the gradient to flow directly between convolutional blocks. This makes backpropagation much more effective in deep architectures.

The training process is performed by minimizing the cross-entropy loss function through gradient descent. The problem when training CNNs is that they require many images (often in the range of millions) to successfully extract relevant features for the final classifier. As most of the time datasets are not large, networks are usually trained on a large dataset (eg, ImageNet [42]) and then fine-tuned on the target dataset. Fine-tuning refers to the process of using an already trained model and retraining it to fit a new distribution. This is much faster because the weights in the model are not randomly initialized, and one can often skip the training of the shallower layers, given the fact that they focus on detecting corners and edges, and thus the model converges faster. Note that the fully connected layer that acts as a classifier in the network must be trained from scratch because the target classes between datasets vary.

For our experiments, we used 101-layer ResneXt [43], which is a residual architecture that uses grouped convolution. This particular architecture uses convolution groups of size 32 with a dimensionality of 8 and a fully connected layer at the end that performs the actual classification. The output of the CNN is a vector that holds the scores for each of the classes; in our case, there were only 2 classes. The network was trained on ImageNet, and we fine-tuned it on our Instagram images. We trained for 8 epochs using stochastic gradient descent with warm restarts [44] with a weight decay of 0.001 and a learning rate of 0.0001 using Nesterov with a momentum of 0.99 on 2 GTX 1080 Ti. We used dropout (50%) to avoid overfitting at the training stage.

#### Classification Tasks

As we wanted to evaluate the change in the performance of models that use 2 different types of control groups, one constituted by users who make use of vocabulary related to suicide (focused control) and another group of users who might not make use of these terms at all (generic control), we created experiments for comparing (1) users at risk versus focused control users (task 1) and (2) users at risk versus generic control users (task 2). These were selected as our 2 supervised predictive tasks. Our instances and their features for the predictive models were previously defined following the process described in the Combining Multimodal Data for Detecting Suicide Risk section.

##### Baselines

We defined as baselines 2 models exclusively based on generic text representations. These models were generated using the previously extracted features and representations from the users: (1) full profile and (2) from their SPV. The first one is a BoW model trained with 1 to 5 grams, and the second one consists of a deep learning model defined by a CNN architecture that has been proven to be successful for text classification [45] and has been used in a similar task that addresses suicide risk assessment on Reddit users [11].

We adopted the approach of Shing et al [11] to define our user-level instances. Therefore, given a user represented by a set of sequential posts, we concatenated all these posts and represented each post as a concatenation of words, where each word is represented by a vector (word embedding), as described in the *Features Definition* section. As in the study by Coppersmith et al [3] and as it has been proven successful on similar tasks, we used a set of word embeddings previously learned on Twitter [35] to define the starting weights for our embedding layer and performed further fine-tuning to learn over the training set and adapt the representations to the task domain.

We considered the 2 models previously described as state-of-the-art approaches for the creation of generic and exclusively text-based models for the task, as it is one of the purposes of our work to analyze the contribution of the additional feature types defined. We therefore defined 4 baseline models. Baselines 1 and 3 correspond to the BoW model generated over the full profile tweets sample and the SPV, respectively. Baselines 2 and 4 correspond to the deep learning model built over the same data samples (full profile and SPV).

##### Classifiers

With the intention of evaluating the individual contribution of the types of features defined, along with their combinations toward a classification/detection task, we considered 4 types of classification algorithms and a deep learning model. We evaluated the performance of random forest, multilayer perceptron, logistic regression, and support vector machines as classifiers. For each feature combination approach, models were built for all these classifiers using the *Scikit-learn* [32] library’s implementation, with a grid search for the best parameters. We used a CNN architecture for the embedding models.

##### Approaches for Combining Features

We evaluated several ways of combining our 3 main feature types defined: generic text-based features, SNPSY features, and the image-based feature (*image user score*). As can be seen in Table 4, we first generated individual models using exclusively all the features corresponding to the *BoW model,* the *embedding’s model,* and the *SNPSY model,* with features mainly obtained from the users’ SPV, as described in Tables 1-3. Afterward, we explored the combination of our different feature types using the BoW model to represent text-based features. Our first approach involves combining the BoW features with the SNPSY features. In this case, given the large number of BoW features and their sparsity, we opted to use the BoW model–predicted probabilities as values for a single feature, denoted as the *BoW outputted feature,* to be added to the SNPSY set of features. This is described in Table 4 as the *BoW+SNPSY model*. Subsequently, we evaluated the combination of the BoW features with the image feature. For this case, we simply added to the BoW set of features the *image user score* as another attribute; this combination is described by the *Images+BoW model*. Afterward, to combine the SNPSY features with the image feature, we used the *image user score* as a new feature in addition to the SNPSY feature set, which is the *Images+SNPSY model*.

Finally, to combine the 3 feature types, we defined 2 approaches. The first approach is an ensemble model where we consider the outputs (predicted probability scores) of the BoW model (*BoW outputted feature)* and SNPSY model (*SNPSY outputted feature*) along with the *image user score*. This approach corresponds to the *Images+BoW+SNPSY model 1* with 3 attributes based on the combination of the 3 independent models with all their features. The second approach consists of using all the features of the SNPSY type as attributes in addition to the *BoW output feature* and the *image user score,* which lead to the definition of *Images+BoW+SNPSY model 2.* It is necessary to recall that the predicted probability scores from the BoW and SNPSY individual models that were used for some of the feature combination approaches at the training stage correspond to the outputs of the classifiers on the test folds during the cross-validation process executed on the training set. This was done to avoid overfitting.

In addition to the combination approaches described, we created 2 other models over which we performed a feature selection procedure over all the feature types. We chose the features with statistically significant differences among the suicide and control groups to evaluate their contribution exclusively to a predictive model. We presented 2 models with features selected based on the *P* values obtained after performing a Mann-Whitney *U* test to compare the samples of each class. This is a feature selection method that has been previously used in medical applications [46]. In addition, we took into account the efficiency of this feature selection approach, given the large feature space considered (Table 4). These models are defined as the *selected features model 1* with the features where *P*<.05, when comparing the suicidal ideation risk and control groups; and the *selected features model 2*, where *P*<.001. The number of features obtained for each model is also given in Table 4.

**Table 4:** Models and features.

Model	Features	Number of features	
		Task 1	Task 2
BoW^a^ model	BoW features generated with the Tf.Idf vectorizer with 1- to 5-gram features	24,645	24,336
Embeddings model	Word embeddings representations as input for a text-based convolutional neural network model	200	200
SNPSY^b^ model	SNPSY features=behavioral+relational+tweets statistics+lexicons+suicide risk factors vocabulary+sentiment analysis features	112	112
BoW+SNPSY model	BoW outputted feature+SNPSY features	24,757	24,448
Images+BoW model	Images user score+BoW features	24,646	24,337
Images+SNPSY model	Images user score+SNPSY features	113	113
Images+BoW+SNPSY model 1	Ensemble model=images user score+BoW outputted feature+SNPSY outputted feature	24,758	24,449
Images+BoW+SNPSY model 2	SNPSY features+images user score+BoW outputted feature	114	114
Selected features model 1	Selected features from all the feature types with *P*<.05	5807	14,882
Selected features model 2	Selected features from all the feature types with *P*<.001	522	3250

^a^BoW: bag of words.

^b^SNPSY: social networks and psychological features.

#### Experimental Setup

##### Dataset Description

Following the description of the Data Collection section, to evaluate our approach, we selected a sample of 252 users with a total of 1,214,474 tweets and 305,637 images, from which up to 1000 images per user were selected for our experiments. We selected a balanced sample of 84 users presenting signs of suicidal ideation (users at risk), 84 focused control users, and 84 generic control users, who were classified within these groups by clinicians after seeing samples of their posts. Table 5 shows the statistics regarding the users belonging to each of the defined groups. We can notice that the median tweet length (in words) is lower for users at risk than for the control and generic cases. We also saw that generic control users produce lower amounts of tweets compared with other types of users.

**Table 5:** Full dataset labeled group statistics.

Description	Suicidal ideation risk group	Focused control group	Generic control group
Number of users	84	84	84
Number of tweets	313,791	766,437	134,246
Median number of tweets per user	2797.5	2984	716
Median tweet length	11	19	14
Number of images	37,801	251,830	16,006

##### Detection Tasks and Evaluation Framework

We considered 3 different aspects to analyze: (1) the utility of having defined the SPV, as we believed that this would allow us to focus on the topic we are analyzing by getting rid of the noise provided by tweets that make no reference to our subject of interest; (2) the individual and combined contribution of the different aspects we analyzed: textual, relational, behavioral, and image-based information; and (3) the change in the performance of models that use 2 different types of control groups, one constituted by users that make use of vocabulary related to suicide (focused control) and another group of generic users who might not make use of these terms at all.

All posts from the full profile of the user were considered for baseline 1 and 2 models, whereas most of the features for our proposed models and combinations were extracted exclusively using the SPV, except for some elements extracted from the user’s tweets metadata and features such as the STTR and DTTR, which required the usage of the posts from the full profile. For each task, 70% of all the instances were retained for training, and the remaining 30% (around 25 users per class) were left for testing purposes as unseen cases. To keep balanced instances from each class, we used stratification for these sets. In addition to these test sets, we also evaluated our best models over a sample of 200 users labeled as doubtful cases. This is done to verify if, as the human annotator, the models are capable of identifying most of these cases as users that are likely to be at risk.

The *PowerTransformer* class from Python’s S*cikit-learn* library was used to transform the feature values to a normal distribution-like representation using Yeo-Johnson’s [32]. To choose the best classifier, a 10-fold cross-validation process was followed over the training set with all the algorithms to evaluate. Afterward, the ones with the best performance were selected to perform a second 5-fold cross-validation along with a grid search to find the most suitable parameters for the classifier chosen.

We considered the Pr, R, F1 score (F1), accuracy, and area under the receiver operating characteristics curve (AUC-ROC) score denoted as AUC, which was the measure on which we based the parameter optimization of the grid search. The values for Pr, R, F1, and AUC corresponded to the suicidal ideation risk class, as it is our main class of interest. We reported on accuracy to analyze the performance of both classes. The results obtained by certain classifiers such as the CNNs were averaged results of multiple runs because of the randomness they can add.

## Results

### Statistical Analysis

We performed an analysis of the features extracted to identify significant differences between the samples of users at risk and our control groups. For each feature extracted for the groups analyzed, we conducted an independent 2-sample Mann-Whitney *U* test among the suicidal ideation group of users and the different control groups. We also conducted this test to compare both of our control groups (focused and generic control groups). We performed a nonparametric test considering that our features do not follow a normal distribution and that there was no homogeneity of variance for most of them.

When comparing the suicidal ideation risk and focused control groups at the SNPSY features, we found significant differences with *P*<.001 among the following features: overall ratio, median time between tweets, verbs, verbs conjugated in singular of the first person (“I”+verb), cognitive mechanisms, anxiety-related terms, usage of personal pronouns, usage of the pronoun “I,” negations, terms to express feelings, and coursing terms. Regarding suicide-related lexicons, the usage of suicide explicit terms, depression-related terms, self-loathing, substance abuse, self-injuries, and terms expressing lack of social support also presented an important significance (*P*<.001). Regarding the features from the BoW model, after conducting the same test, we found significant differences with *P*<.001 for n-grams such as *I feel, sad, kill myself, cry/crying, depression, to die, horrible, anxiety, die, pills*, among others*.* Considering all the features used (24,758), a total of 522 features were significant for distinguishing the groups according to these tests with *P*<.001. Table 6 shows the medians and the distributions overlapping index [47] for both groups on a sample of relevant features.

When repeating the independent two-sample Mann-Whitney *U* test to compare the suicidal ideation risk group with the generic control set of users regarding the SNPSY features, among the ones with *P*<.001, we found the median classifier score, the number of tweets generated, and the median time between tweets to be different among both groups (suicidal ideation risk vs generic control). We identified differences in discussion topics such as money and work, about which the generic control users seem to discuss more, whereas the members of the suicidal ideation risk group use terms more related to health and biological aspects. As in the previous case, the use of self-references was higher in the suicidal ideation risk group. Within the significant n-grams from the BoW model, we found terms such as *feel, to die, songs, someone, cry/crying, anxiety, life, breath, bad, and fear*. This is shown in Table 7, which displays the median value and overlapping index of the distributions of the groups in terms of some of the attributes mentioned. Again, taking into account all the features used (24,449), 3250 were significant for distinguishing between the suicidal ideation risk and the generic control group in terms of this test with *P*<.001.

Regarding other features explored, considering a 95% CI, for *task 1* (suicidal ideation risk vs focused control), the number of friends (*P*=.04) and median tweet length (*P*=.04) were significantly different. For these cases, the median number of friends for a focused control user (578.5) was higher than the median number of friends at risk (372.0). The same was true for the median tweet length, based on the SPV, which was higher for focused control users with 16 words against 13 of the suicidal ideation risk users. In addition, there were significant differences in the STTR (*P*=.049) and weekday count ratio (*P*=.01). Under the same CI, for *task 2,* the weekday count ratio (*P*=.001), the STTR (*P*=.004), along with the number of followers (*P*=.05), and the total amount of favorites given (*P*=.006) showed significant differences. In this sense, generic control users appeared to tweet more on weekdays (Monday to Friday) as well as focused control users, whereas the opposite behavior was found for suicidal ideation risk users. Regarding the median STTR, generic control users obtained an STTR value of 0.02, whereas users at risk obtained an STTR value of 0.04, meaning that users at risk seemed to tweet more at night compared with the generic and focused control users as well.

The image scores were also significantly different according to the test with *P*=.002 for the comparison between the suicidal ideation risk and generic control groups, considering a 95% CI. Curiously, for the comparison of the image scores between the suicidal ideation risk group and the focused control group, the test scores were different, with *P*=.05. This can be explained by the fact that users providing information or news about suicide make use of similar images, which characterize the condition, making it difficult to find a significant difference only judging by pictures. As can be seen in Table 8, for both the control groups and the suicidal ideation risk group, the median image scores were slightly higher for the suicidal ideation risk group.

Finally, to compare our control groups (focused and generic control groups), we performed the same test (Mann-Whitney *U* test) and found significant differences between some of these groups’ features (n=181) with *P*<.001. Among these features, we found mainly suicide-related lexicons, such as suicide methods, suicide explicit terms, bullying, discrimination, and substance abuse–related terms. We also found differences (*P*<.001) in other textual, relational, and behavioral attributes, such as the number of tweets, number of friends, number of followers, median favorites and retweet counts, overall ratio, polarity score, median time between tweets, and STTR, among others. These differences confirmed our previous assumptions regarding the differences among the control groups.

**Table 6:** Medians and Distribution Overlapping Index for some of the attributes with the most significant differences between the Suicidal ideation and Focused control groups.

Attribute	Suicidal ideation median	Focused control median	Overlapping index
Anxiety	10.94	0	0.25
Coursing terms	21.52	7.68	0.43
To die (self-reference)	5.45	0	0.25
I feel	46.25	6.71	0.32
Self-loathing	0.03	0	0.35
Verb I	22.66	12.11	0.41

**Table 7:** Medians and Overlapping Index for some of the attributes with the most significant differences between the Suicidal ideation and Generic control groups.

Attribute	Suicidal ideation median	Generic control median	Overlapping index
Median classifier score	0.72	0.65	0.46
To die	19.5	0	0.25
Number of user tweets	2076.5	453	0.38
Health	17.19	8.18	0.44
Work	35.46	49.59	0.44
I	41.32	9.60	0.23

**Table 8:** Medians and Overlapping Index for the images score between the suicidal ideation, focused control and generic control classes.

Attribute	Group	Median value	Overlapping index
Images score	Suicidal ideation	0.24	0.64
	Focused control	0.23	
	Suicidal ideation	0.24	0.52
	Generic control	0.23	

### Classification Task Results

In this section, we reported the results of our experiments. Table 9 presents the evaluation measure results for each task on the test sets. We reported the results for the best models, as described in Table 4, along with the baselines.

**Table 9:** Predictive task results.

Model	Suicidal ideation versus focused control group							Suicidal ideation versus generic control group					
	Pr^a^	R^b^	F1^c^	Ac^d^	AUC^e^	Classifier	Pr		R	F1	Ac	AUC	Classifier
BoW^f^ model—full profile (baseline 1)	0.78	0.81	0.79	0.78	0.81	MLP^g^	0.79		0.85	0.81	0.80	0.91	MLP
Embeddings model—full profile (baseline 2)	0.76	0.81	0.79	0.77	0.82	CNN^h^	0.78		0.87	0.82	0.80	0.84	CNN
BoW model—SPV^i^ (baseline 3)	0.81	0.85	0.83	0.82	0.85	LR^j^	0.80		0.92^k^	0.86	0.84	0.89	MLP
Embeddings model—SPV (baseline 4)	0.79	0.85	0.82	0.80	0.83	CNN	0.77		0.87	0.82	0.80	0.82	CNN
SNPSY^l^ model	0.85	0.85	0.85	0.84	0.86	SVM^m^	0.85		0.88	0.87	0.86	0.94	LR
BoW+SNPSY model	0.82	0.88^k^	0.85	0.84	0.89	RF^n^	0.85		0.88	0.87	0.86	0.94	LR
Images+BoW model	0.79	0.88^k^	0.84	0.82	0.86	MLP	0.82		0.88	0.85	0.84	0.90	LR
Images+SNPSY model	0.88^k^	0.85	0.86^k^	0.86^k^	0.91	SVM	0.88		0.88	0.88^k^	0.88^k^	0.94	LR
Images+BoW+SNPSY model 1	0.85	0.85	0.85	0.83	0.87	LR	0.85		0.92^k^	0.88^k^	0.88^k^	0.92	MLP
Images+BoW+SNPSY model 2	0.88^k^	0.81	0.84	0.84	0.92^k^	SVM	0.85		0.88	0.87	0.86	0.94	LR
Selected features model 1 (*P*<.05)	0.85	0.85	0.85	0.84	0.90	MLP	0.91^k^		0.77	0.83	0.84	0.94	SVM
Selected features model 2 (*P*<.001)	0.83	0.77	0.80	0.80	0.92^k^	SVM	0.91^k^		0.81	0.86	0.86	0.95^k^	SVM

^a^Pr: precision.

^b^R: recall.

^c^F1: F1 score.

^d^Ac: accuracy.

^e^AUC: area under the curve.

^f^BoW: bag of words.

^g^MLP: multilayer perceptron.

^h^CNN: convolutional neural network.

^i^SPV: short profile version.

^j^LR: logistic regression.

^k^The best results for each of the evaluation measures.

^l^SNPSY: Social networks and psychological features.

^m^SVM: support vector machine.

^n^RF: random forest.

#### Short Profile Version Definition Results

As can be seen in Table 9, the definition of the SPVC is successful as the first filter for both the predictive tasks. Indeed, the BoW models trained exclusively on the SPV (baselines 3 and 4) outperformed baselines 1 and 2 for most of the measures on both tasks. For these representations, tweets unrelated to the topic seem to introduce noise, as they generate a bigger feature space. In contrast, setting a high decision threshold for the classifier implies reducing the vocabulary for the BoW model, which might reduce the performance of the model with the test data.

Regarding the CNN embedding models trained exclusively on the SPV, we can see that the model of task 1 obtains slightly better results compared with the baseline 2 model, whereas the results do not differ much for task 2. In general, we observed a better performance with the SPV for BoW models. Therefore, the combinations evaluated take into account these text-based representations (BoW).

It is important to recall that for the focused control cases, after applying the SPVC with a decision threshold over 0.5, 4 users were left without an SPV because any of their tweets obtained a predicted probability over the threshold. Considering that with higher thresholds, more focused control and generic users could be lost for training our next classifier, this is the threshold we kept for our further experiments (0.5). However, these results also showed that using SPVC reduces the number of control users with an SPV as the threshold value rises and that the definition of the SPV is useful for discarding users who do not present tweets similar to those of the users at risk. Initially, we found that focused control users were more easily discarded than generic users. However, this could be explained by the fact that the control users discarded might correspond to informative accounts such as newspapers, which we assumed to make use of certain terms referring to suicide in a way that does not make use of terms that imply a personal reference or opinion; therefore, the first classifier might find it easier to discard. In any case, this is a supposition as we did not have further access to the writings of users after the annotation.

#### Combining Model Results

Regarding the methods considered for combining the types of features extracted, we can observe that when these types are evaluated independently from each other, each has a good accuracy, with the SNPSY model obtaining the best results. For the combinations reported in Table 4 for the suicidal ideation risk versus focused control groups, we can observe that the models that use the 3 types of features do not significantly improve the results obtained by the SNPSY model. However, for Images+BoW+SNPSY model 2, we can see a 7% and 11% increase in the AUC score compared with baseline 3 and baseline 1, respectively, for the suicidal ideation versus focused control cases. The AUC difference of their ROC curves using the Delong method was *P*=.04, which is statistically significant, considering a 95% CI.

For task 2, the Images+BoW+SNPSY combination obtained results that improved baseline 1 for the suicidal ideation versus generic control task. For the Images+BoW+SNPSY model 1, we noticed a 4% increase in accuracy compared with baseline 3, and it increased to 8% compared with baseline 1. There was also an increase in the AUC value of up to 4% with the selected features model 2. We also noticed the same measured results between the SNPSY model, the BoW+SNPSY model, and the Images+BoW+SNPSY model 1, implying that we might not improve the performance of the SNPSY model by adding other feature types. In fact, after conducting a Delong test to compare the ROC curves of these models with the baseline 1 model, we could not find significant differences, implying that their performance was not significantly different from the baseline in terms of the AUC measure for this task. However, this also implied that the use of the SNPSY features alone allowed us to have a model with a reduced number of features that perform as well as the BoW model with thousands of features.

Regarding the role of the images, we can see that when they are individually combined either with the BoW features or the SNPSY features, either the F score or the AUC score increases minimally compared with baseline 3. As part of the experiments for this approach, it is necessary to mention that as some image scores were missing for a few users (up to 4 for each task), the approach considered to address this issue was to replace the scores by the mean of all the users except for the model where only a single score for each feature type was considered; for this case, the instances with missing values were removed.

In reference to the models with a set of selected features, we can notice that these models also outperform baselines 1 and 2 in terms of F1, accuracy, and AUC. The selected features model 1 for both tasks outperformed baselines 3 and 4 on F1 and AUC. It should be noted that these models consider a reduced number of features compared with the baseline models, and the Images+BoW model, as they attempted to reduce the overfitting that the usage of thousands of features might imply.

It is important to mention that for the models evaluated; we did not address the definition of specialized decision thresholds for the classifiers. Therefore, for future work, this should be considered because false-negative predictions should be minimized, and a threshold should be defined to maximize the R value of the suicidal ideation class without leaving aside the tradeoff with Pr. Regardless of this, we can see that most of our models outperformed the baselines in terms of the AUC score, which implies that our models are capable of distinguishing between the classes successfully. In this sense, the definition of a better decision threshold can only contribute to improving the performance of our models.

#### Comparative Results of Tasks

When comparing the results of both tasks, we saw that the results obtained by the models to distinguish between users at risk from generic control users were not that different from those trained over focused control users. However, we noticed always higher levels of certainty for the models trained to compare users at risk and generic control users. This can be observed when comparing the AUC scores, which are always higher for the models of task 2. In fact, for this task, we can see that a high AUC score is already obtained by the baseline models, and it does not improve significantly with other models. This differs from task 1, where the feature combination is relevant for improving the certainty of the models compared with the baseline.

**Figure 1:**
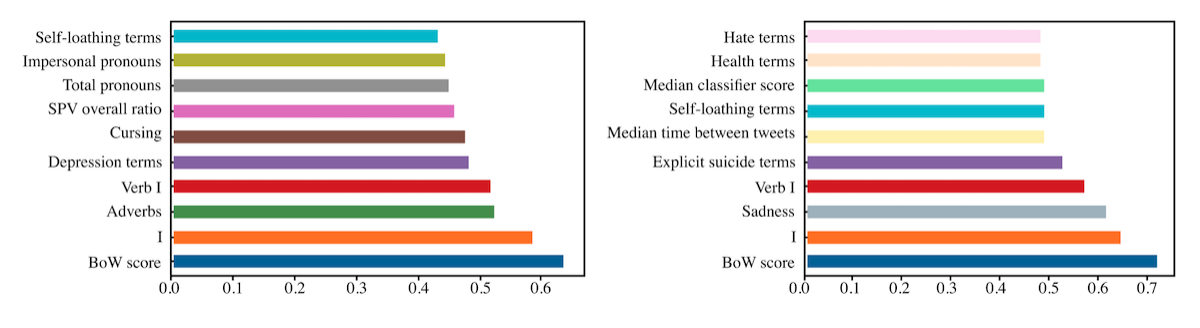
Features more correlated with the class to predict for both tasks: Suicidal ideation risk vs Focused control (left), and Suicidal ideation risk vs Generic control (right).

Figure 1 shows the top 10 most correlated features with the class for each task considering the features of Images+BoW+SNPSY model 2. The most correlated features were given by textual elements such as the BoW model scores and lexicons. It is interesting to see that a behavioral feature as the median time between tweets is relevant for task 2. We can also notice that for both tasks, self-references are relevant and that the usage of explicit suicide terms and health-related terms is relevant for task 2, as generic control users are not characterized by the usage of terms related to suicide.

**Figure 2:**
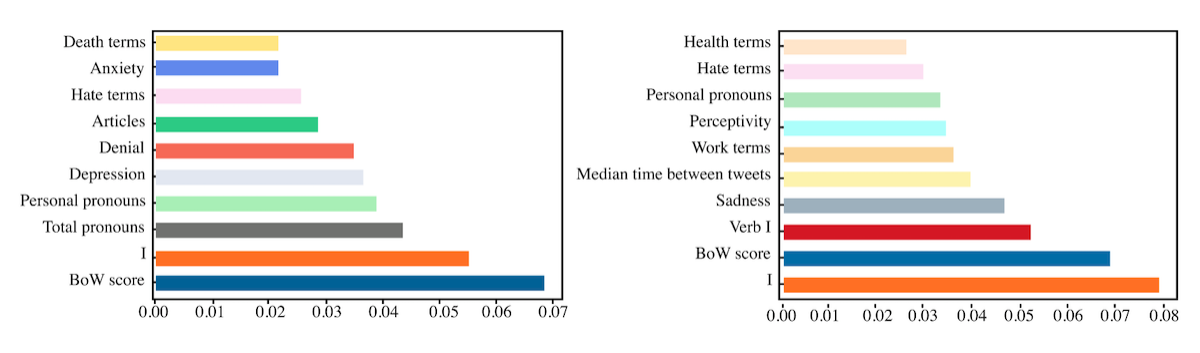
Most predictive features for both tasks: Suicidal ideation risk vs Focused control (left), and Suicidal ideation risk vs Generic control (right).

Referring to the features that were more predictive for the models generated, we considered the random forest’s feature importance function, which is based on its measure of impurity. In this sense, we can see how much each feature decreases the impurity. The more a feature decreases the impurity, the more important is the feature. In this case, because random forest uses multiple trees, the impurity decrease from each feature was averaged across all trees to determine the final importance of the variable. The most important features based on this approach, considering the features of Images+BoW+SNPSY model 2 is shown in Figure 2. For this case, we confirmed that for task 2, the usage of terms related to work and health is distinctive for both classes, as mentioned in the *Statistical Analysis* section.

For both approaches, we can see that the image scores do not appear within the features more relevant for the tasks, implying that textual and behavioral features can be more relevant. Regardless of this, the scores given by certain feature combinations showed that the inclusion of the image scores improves minimally the results of these predictive tasks. Some of the images that most activated neurons at the image processing stage involve pictures of people crying and images containing the term suicide written within them.

We also evaluated the *selected features model 2*, as one of our models with the best results for AUC for both tasks, over a sample of 200 users who were initially labeled as *doubtful* cases. We evaluated 2 models, one trained with the data of task 1 (selected features model 2—task 1) and another trained with the data of task 2 (selected features model 2—task 2). For the first model, we predicted 65% of the doubtful cases as positive (risk), whereas for the second model, 73% of the doubtful cases were found to be at risk. This indicates that our models detected signs of suicidal ideation in more than half of the doubtful users, which is in concordance with the criteria of the first annotator.

Finally, we evaluated the *selected features model 2—Task 1* over a test set of suicidal ideation and generic control users to evaluate the performance of this model over users who do not use a suicide-related vocabulary. We obtained the following results: Pr=0.91, R=0.77, F1=0.83, accuracy=0.84, and AUC=0.95. These results showed that the model obtains better results than generic control users in comparison with its performance over focused control users. Similarly, we evaluated *the selected features model 2—task 2* over a test set of suicidal ideation and focused control users obtaining Pr=0.83, R=0.80, F1=0.82, accuracy=0.82, and AUC=0.91. The performance of this model was worse than that of generic control users and was consistent with the fact that distinguishing these 2 cases is much harder.

## Discussion

### Principal Findings

This study presented a methodology for suicide risk assessment on social media. We extracted information from multimodal data to build statistical and deep learning–based predictive models. Our models consider a set of features based on BoW and n-grams, lexicons, relational, statistical, and behavioral information, in addition to an image analysis. To the best of our knowledge, this is the first approach that addresses the combination of all these types of features for suicide risk assessment at the user level. Moreover, we highlighted the usefulness of discarding the noise of writings not related to the topic of study through the definition of an SPV, which outperforms the baseline given by the analysis of the full profile of the user, with an increase in accuracy and F1.

We also compared the performance of predictive methods trained on different control groups with the goal of making a more specialized classifier capable of distinguishing users at risk from control cases, even when the discussed topic is similar. Better results are achieved in terms of AUC-ROC when using generic control users instead of users who make use of suicidal vocabulary. We also highlighted the importance of the interpretability of our features, considering elements that can be understood by clinicians and mapped to their screening practice. The results of our experiments showed that within the types of features analyzed, there are multiple significant features that may lead to the detection of risk situations, the most relevant ones are based on the identification of textual and behavioral elements such as self-references, the number of tweets posted, and the time that passes between each post (*P*<.001).

Text-based features were the most relevant for our model; however, their combination with image-based scores, along with relational and behavioral aspects, allowed us to obtain models that outperform the results provided by an exclusively text-based model.

### Limitations

Our study presents some limitations, given mainly by the fact that this is an observational study where there is no access to the personal and medical information that is often considered in risk assessment studies. Limitations are given by the accuracy of the methods applied to infer some of these aspects based on the vocabulary and behavioral patterns of the users. For instance, the weekend tweets count ratio is because of the difference in the posting time according to the user’s time zone and the sleeping time tweets ratio, for which we assume that the sleeping time is the period in a day when the user has less activity.

A representativeness analysis regarding age, gender, and location of the users analyzed was not performed, considering that this type of information is not available on Twitter. However, the biases existing in our data samples, regarding these aspects, follow similar gender and age biases for suicides [48], as most Twitter users are male and middle aged [14]. In addition to this, the fact that the lexicon used to extract data was obtained from a different source such as Reddit might imply biases introduced by the type of vocabulary used in Reddit, along with the limitations that the translation to Spanish might introduce even if the terms and phrases used were thoughtfully inspected and verified by specialized clinicians.

Finally, there are also limitations given by the nature of the users who post on Twitter, as they might differ from users at risk who do not choose to make their profiles public or even from users at risk who do not have a Twitter account. In addition, it is not guaranteed that the users annotated as users at risk are actually at risk because the annotation was performed just from reading a few tweets.

### Reproducibility and Ethical Concerns

The analysis of data provided by social networks to detect health problems and assist clinicians is an open issue, not uncontroversial. The aim of our proposal, however, is to shed light on the real capabilities of these systems in a specific theoretical application: suicide risk prevention. Before such systems become available, a careful risk-benefit assessment along with a proper analysis of applicable legal framework compliance and the potential threats to users’ privacy and civil liberties shall be conducted [12,26,49].

On the reproducibility of this work, we should respect Twitter's policies on the distribution of the data collected through its API. Taking into account the restrictions of sharing any information that can lead to identifying the users of our study [12], only the calculated features of our experiments along with the code and parameters used on the classifiers will be available under request with a proper explanation of the usage intended for the models. Finally, it is also necessary to address the potential scenarios of misusage of tools based on these models. Guntuku et al [9] mentioned hypothetical cases where these types of screening tools can be used by employers or insurance companies against the interests of people with mental disorders; therefore, policies against these applications shall be defined before their release.

### Future Work

By observing the performance of image-based predictive models, we believe that our results can be improved by enhancing the contribution of the textual and relational features. This can be done by exploring other text representation methods and analyzing deeply the network and interactions between users. In addition, we believe that these methods can be extended and applied to different use cases related to mental disorders, such as depression, anxiety, or eating disorders.

We believe that the approach presented in this study can evolve into a real-time system that emits alerts when users at risk are found. It is necessary to recall that given that scenario, the idea of such a tool is to be the first filter to assist clinicians and does not intend by any means to replace their work. In fact, a readable subset of tweets from the SPV, with the top *k* tweets ordered according to the SPVC, can be an output of the system for clinicians to evaluate and proceed with future screening steps, if allowed. Indeed, legal and ethical issues for the deployment of such systems should be analyzed before. However, we believe that a tool of this kind can be the starting point for the development of noninvasive interventions where specialists and social media community members can contribute to the recovery and prevention of mental health issues and suicide. Again, the appropriate protocols and procedures should be defined for this instance. In particular, there should be a deep analysis of the implications of a potential intervention taking into account the legal boundaries set for the treatment of personal data in this context.

## Abbreviations

API: application programing interface
AUC: area under the receiver operating characteristics
AUC-ROC: area under the receiver operating characteristics curve
BoW: bag of words
CNN: convolutional neural network
DTTR: daytime tweets ratio
LIWC: Linguistic Inquiry and Word Count
SNPSY: social networks and psychological features
SPV: short profile version
SPVC: short profile version classifier
STTR: sleep time tweets ratio
